# Book Reviews

**Published:** 1894-02

**Authors:** 


### Book Reviews.

HERNIA: Its Palliative and Radical Treatment in Adults, Children and Infants. By THOMAS H. MANLEY, A. M., M. D., Visiting Surgeon to Harlem Hospital; Consulting Surgeon to Fordham Hospital; Member of the New York Academy of Medicine, American Medical Association, New York State and County Medical Associations, International Medical Congress, Pathological Society, National Association of Railway Surgeons, etc., etc. Octavo, pp. 231. Philadelphia: The Medical Press Co., L'td. 1893.

An inspection of this book does not convince us that it contains enough that is new, or even that is better said than in the many works on hernia which are now accessible to the medical profession, and, hence, we do not see that it will do much else than announce the existence of the author. This does not mean that Manley's treatise has not some good features. There is much sound sense in what the author has to say on the subject of trusses in infancy, but the general plan adopted by him does not show the consistency and appreciation of the relative importance of the various portions of the subject that one has a right to expect in a book placed before the profession, the majority of whose readers are not competent to separate the wheat from the chaff. In short, the inference is clear that the writer is not a teacher or experienced as an author.

For instance, on pages nine and ten it is stated that "a general impression prevails that the detection of hernia, its diagnosis and recognition, are simple." This is anything but correct. Thus, in a few words replete with tautology, the author affirms an opinion that he evidently believes worthy of emphasis, and yet, one may hunt his book through without finding anything tending to throw light upon the difficulties acknowledged. Again, he asserts that "we must catch the hare before we can cook him." This particular hare does not get cooked, because he is still running when the book is concluded.

Still again, a considerable improvement can be made in the construction of sentences, such as, for example, "if we wish to cocainize without inflicting, but the minimum of pain, etc." (p. 119), and the use of terms like "herniated arca," "hypodermize tissues," "hypodermication," and the like. Until one can write grammatically, he has no right to pose as the author of a book intended for circulation only among the members of an educated and scientific profession.

Another peculiarity to be found in this book is the method of reaching conclusions. We need only call attention to the author's remarks upon the Kocher operation (pp. 181–182), where in a few paragraphs one will find all the rubbish that could possibly be condensed into such a measure of space, and, to cap the climax, after stating the most positive (the author says fundamental) objections to the method, he admits that these objections "may be and rather visionary than practical."

Our advice to the author is to wield the knife and not the pen. With the former we have no doubt he is judicious and skilful; with the latter he can only bring ridicule upon himself and undo a reputation as a surgeon that he may really deserve.

J. P.

---

New Truths in Ophthalmology: As Developed by G. C. Savage, M. D., Professor of Ophthalmology in the Medical Department of the University of Nashville and Vanderbilt University. With thirty-two illustrations. Small 8vo, pp. viii.—152. Published by the Author. Price, $1.50. Nashville, Tenn. 1893.

In the main, this book is a collection of the principal contributions which the author has made to ophthalmic literature during the past five years. Muscular anomalies of the eyes, and refractive errors and their correction, occupy the larger part of the work. That portion treating of the insufficiencies of the oblique muscles is especially interesting, and traverses a field heretofore unexplored. "Rhythmic exercise" of the ocular muscles is also of interest, and is worthy of practical consideration. The author writes in that forcible and earnest style native to himself, which impresses the reader with the genuineness and strength of his convictions; and while all that is here presented as *truth* may not be accepted fully by others, yet there is so much that is instructive and so much that is pertinent to the hour, that all interested in anomalies of the ocular muscles, and of refraction, should read the book.

A. A. H.

### BOOKS RECEIVED.

International Clinics. A Quarterly of Clinical Lectures on Medicine, Neurology, Pediatrics, Surgery, Genito-Urinary Surgery, Gynecology, Ophthalmology, Laryngology, Otology, and Dermatology. By professors and lecturers in the leading medical colleges of the United States, Great Britain and Canada. Edited by John M. Keating, M. D., LL.D., Colorado Springs, Col.; Fellow of College of Physicians, Philadelphia ; formerly Consulting Physician for Diseases of Women to St. Agnes' Hospital ; Gynecologist to St. Joseph's Hospital ; Visiting Obstetrician to the Philadelphia Hospital, and Lecturer on Diseases of Women and Children, Philadelphia ; Editor Cyclopedia of the Diseases of Children. Judson Daland, M. D., Philadelphia, Instructor in Clinical Medicine, and Lecturer on Physical Diagnosis and Symptomatology, in the University of Pennsylvania ; Assistant Physician to the University Hospital ; Physician to the Philadelphia Hospital and to the Rush Hospital for Consumption. J. Mitchell Bruce, M. D., F. R. C. P., London, England, Physician and Lecturer on Therapeutics at the Charing Cross Hospital. David W. Finlay, M. D., F. R. C. P., Aberdeen, Scotland, Professor of Practice of Medicine in the University of Aberdeen ; Physician to, and Lecturer on, Clinical Medicine in the Aberdeen Royal Infirmary ; Consulting Physician to the Royal Hospital for Diseases of the Chest, London. Volume II. Third series, 1893. Royal octavo, pp. xii.—363. Philadelphia : J. B. Lippincott Co. 1893.

Twelfth Annual Report of the State Board of Health of New York. Transmitted to the Legislature, February, 1892. Octavo, pp. 558. Albany : James B. Lyon, State Printer. 1892.

Thirteenth Annual Report of the State Board of Health of New York. Transmitted to the Legislature, March 9, 1893. Octavo, pp. 736, with maps. Albany : James B. Lyon, State Printer. 1893.

Proceedings of the Sanitary Convention, held at Stanton, April 27 and 28, 1893. Supplement to the Report of the Michigan State Board of Health for the year 1893. Lansing : Robert Smith & Co., State Printers and Binders.

State Board of Health of New York. Local Boards of Health in the State of New York. Albany : The Argus Company, Printers. 1893.

An American Text-Book of Gynecology, Medical and Surgical. For the Use of Students and Practitioners. By Henry T. Byford, M. D., John M. Baldy, M. D., Edwin Cragin, M. D., J. H. Etheridge, M. D., William Goodell, M. D., Howard A. Kelly, M. D., Florian Krug, M. D., E. E. Montgomery, M. D., William R. Pryor, M. D., George M. Tuttle, M. D. Edited by J. M. Baldy, M. D. Forming a handsome royal octavo volume, with 360 illustrations in text, and thirty-seven colored and half-tone plates. Price, cloth, $6.00 ; sheep, $7.00 ; half Russia, $8.00. Pp. xxiv.—713. For sale by subscription. Philadelphia : W. B. Saunders, 925 Walnut street. 1894.

Manual of Physical Diagnosis for the Use of Students and Physicians. By James Tyson, M. D., Professor of Clinical Medicine in the University of Pennsylvania, and Physician to the University Hospital ; Physician to the Rush Hospital for Consumption and Allied Diseases ; Fellow of the College of Physicians of Philadelphia ; Member of the Association of American Physicians, etc. Second edition, revised and enlarged. Duodecimo, pp. 241. Philadelphia : P. Blakiston, Son & Co., 1012 Walnut street. 1893.

An Outline of the Embryology of the Eye. With illustrations from original pen drawings by the author. By Ward A. Holden, A. M., M. D., Assistant Surgeon New York Ophthalmic and Aural Institute; Clinical Assistant Vanderbilt Clinic. The Cartwright Prize Essay for 1893. Duodecimo, pp. 69. New York : G. P. Putman's Sons, 27 West Twenty-third street. London : The Knickerbocker Press, 24 Bedford street, Strand. 1893.

How to Use the Forceps, with an Introductory Account of the Female Pelvis and of the Mechanism of Delivery. By Henry G. Landis, A. M., M. D., Professor of Obstetrics and Diseases of Women and Children in Starling Medical College, Columbus, O. Revised and enlarged by Charles H. Bushong, M. D., Assistant Gynecologist and Pathologist to Dewitt Dispensary, New York. Small 8vo, pp. 203; illustrated. New York : E. B. Treat, Publisher, 5 Cooper Union. 1894. Price, $1.75.

A Practical Treatise on Nervous Exhaustion (Neurasthenia) : Its Symptoms, Nature, Sequences, Treatment. By George M. Beard, A. M., M. D., Fellow of the New York Academy of Medicine, of the New York Academy of Sciences; Vice-President of the American Academy of Medicine; Member of the American Neurological Association, of the American Medical Association, the New York Neurological Society, etc. Edited, with notes and additions, by A. D. Rockwell, A. M., M. D., Professor of Electro-Therapeutics in the New York Post-Graduate Medical School and Hospital; Fellow of the New York Academy; Member of the American Neurological Association, of the NewYork Neurological Society, etc. Third edition, enlarged. Small 8vo, pp. 262. New York: E. B. Treat, 5 Cooper Union. 1894. Price, $2.75.

Annual Report of the Postmaster-General, of the United States, for the fiscal year, ending June 30, 1893. Octavo, lix—744. Washington: Government Printing Office. 1893.

A Manual of Diseases of the Nervous System. By W. R. Gowers, M. D., F. R. C. P., F. R. S., Consulting Physician to University College Hospital; Physician to the National Hospital for the Paralysed and Epileptic. Second edition, revised and enlarged. Volume II. Diseases of the Brain and Cranial Nerves, General and Functional Diseases of the Nervous System. With 182 illustrations, including a large number of figures. Octavo, pp. xvi—1069. Philadelphia: P. Blakiston, Son & Co., 1012 Walnut street. 1893.

A Manual of Practical Hygiene. Designed for Sanitary and Health Officers, Practitioners and Students of Medicine. By W. M. Coplin, M. D., Adjunct Professor of Hygiene, Demonstrator of Pathology, and Curator of the Museum, Jefferson Medical College; Adjunct Professor of Pathology in the Philadelphia Polyclinic and College for Graduates in Medicine; Surgeon to St. Mary's Hospital; Pathologist to the Philadelphia Hospital; late A. A. Surgeon, U. S. Marine Hospital Service; and D. Berau, M. D. Instructor in Hygiene and Clinical Microscopy, Jefferson Medical College; Bacteriologist to St. Agnes' Hospital, Philadelphia; Assistant Pathologist, Philadelphia Hospital; Assistant Pathologist, Philadelphia Polyclinic and College for Graduates in Medicine. With an introduction by H. A. Hare, M. D., Professor of Therapeutics, Materia Medica, and Hygiene in Jefferson Medical College, Philadelphia. With 140 illustrations, many of which are printed in colors. Octavo, pp. xvi—456. Philadelphia: P. Blakiston, Son & Co., 1012 Walnut street. 1893.

